# Identification and Molecular Mechanism of Novel α-Glucosidase Inhibitory Peptides from the Hydrolysate of Hemp Seed Proteins: Peptidomic Analysis, Molecular Docking, and Dynamics Simulation

**DOI:** 10.3390/ijms26052222

**Published:** 2025-02-28

**Authors:** Zhang Mengyuan, Chen Chen, Wei Feng, Zhao Ning, Yang Wanyu, Zhang Tianrong, Ren Guoyan, Qiu Zhijun, Zhang Bin

**Affiliations:** 1 College of Food and Bioengineering, Henan University of Science and Technology, Luoyang 471023, China; mengyuanzhang0127@163.com (Z.M.); xichen3820@163.com (C.C.); weifeng2002025@163.com (W.F.); 230320070641@stu.haust.edu.cn (Y.W.); zhangtianrong1002@163.com (Z.T.); renguoyan@163.com (R.G.); qiuzj2003@163.com (Q.Z.); 2 Academy of Military Medical Sciences, Beijing 100850, China; zhaoning19901009@126.com; 3 Henan Engineering Research Center of Food Microbiology, Luoyang 471023, China; 4 National Demonstration Center for Experimental Food Processing and Safety Education, Luoyang 471023, China

**Keywords:** α-glucosidase inhibitory peptides, hemp seed proteins, in silico analysis, peptidomics, molecular docking, molecular dynamics

## Abstract

There is a growing demand for natural and potent α-glucosidase inhibitors due to the rising prevalence of diabetes. In this study, newly identified α-glucosidase inhibitory peptides were identified from the tryptic hydrolysate of hemp seed proteins based on peptidomics and in silico analysis. A total of 424 peptides, primarily derived from four cupin-type-1 domain-containing proteins, were identified, and 13 ultimately were selected for validation based on their higher PeptideRanker scores, solubility, non-toxicity, and favorable ADMET properties. Molecular docking revealed that these 13 peptides primarily interacted with α-glucosidase via hydrogen bonding and hydrophobic interactions. Among them, three novel peptides—NPVSLPGR (−8.7 kcal/mol), LSAERGFLY (−8.5 kcal/mol), and PDDVLANAF (−8.4 kcal/mol)—demonstrated potent α-glucosidase inhibitory activity due to their lower binding energies than acarbose (−8.1 kcal/mol), the first approved α-glucosidase inhibitor for type 2 diabetes treatment. The molecular mechanism analysis revealed that the peptides NPVSLPGR and LSAERGFLY inhibited α-glucosidase by simultaneously blocking substrate entry through occupying the entrance of the active site gorge and preventing catalysis by binding to active sites. In contrast, the peptide PDDVLANAF primarily exerted inhibitory effects by occupying the entrance of the active site gorge. Molecular dynamics simulation validated the stability of the complexes and provided additional insights into the molecular mechanism determined through docking. These findings contribute essential knowledge for the advancement of natural α-glucosidase inhibitors and offer a promising approach to effectively manage diabetes.

## 1. Introduction

The global prevalence of diabetes has gradually increased in recent decades due to improved living conditions and lifestyle changes, such as excessive calorie intake, lack of exercise, and late-night habits. According to International Diabetes Federation (IDF), in 2021, an estimated 537 million people worldwide were affected by diabetes, with a prevalence of 10.5%. This number is expected to rise to 783.2 million by 2045, corresponding to a prevalence of 12.2% [1]. Diabetes can cause significant damage and dysfunction to organs and tissues, leading to progressive metabolic complications. These complications further contribute to increased morbidity and mortality, shortened life expectancy of patients and substantial economic burden on both society and individuals [2]. Diabetes is classified into type 1, type 2, and gestational diabetes, with type 2 diabetes (T2DM) accounting for approximately 80% of all cases. T2DM is characterized by hyperglycemia caused by improper insulin secretion and regulation, resulting in elevated blood glucose levels that exceed safe thresholds [3]. In terms of the treatment of T2DM, strategies that prevent carbohydrate digestion, such as the use of α-glucosidase inhibitors, are widely adopted. These inhibitors are recommended by IDF as first-, second-, and third-line treatment options [4].

Acarbose, a naturally derived pseudo-oligosaccharide, was the first α-glucosidase inhibitor approved for the treatment of T2DM [5]. Despite its therapeutic efficacy and low cost, the use of acarbose is associated with several side effects, including flatulence, diarrhea, abdominal distention, and nausea, as demonstrated in clinical trials [6]. Alternatively, recent studies have suggested that bioactive peptides possess a wide range of biological activities, with α-glucosidase inhibitory activity being particularly notable for its potential role in the prevention and management of T2DM. In this regard, finding peptides with potent α-glucosidase inhibitory activity from natural resources has become a new way to address the side effects of existing α-glucosidase inhibitors.

A large number of α-glucosidase inhibitory peptides derived from various food sources have been reported in recent studies. For example, the hydrolysate prepared from the hot-pressed peanut meal using the combination of Alcalase and Neutrase demonstrated favorable α-glucosidase inhibitory activity with an IC_50_ of 5.63 ± 0.19 mg/mL. From the hydrolysate, four novel α-glucosidase inhibitory peptides, i.e., FYNPAAGR, PGVLPVAS, FFVPPSQQ, and FSYNPQAG, were identified by nano-LC-MS/MS, and their inhibitory mechanisms were analyzed through molecular docking [7]. Similarly, many other plants are also reported as the resources for α-glucosidase inhibitory peptides, such as sweet potato [8], soy [9], *Ginkgo biloba* [10], and foxtail millet [11]. On the other hand, animal proteins are also attractive for discovering novel α-glucosidase inhibitory peptides. For instance, four peptides, including LPFQR, PSFD, PSFDF, and VPFPR, were identified from the defatted Antarctic krill powder serially by optimized hydrolysis with Neutrase, gel chromatography, and LC-MS/MS techniques. These peptides exert their α-glucosidase inhibitory activities by binding strongly to the active sites of α-glucosidase via hydrogen bonding and hydrophobic interactions [12]. Additionally, other animal proteins, such as soft-shelled turtle egg [13], *Andrias davidianus* collagen [14], and Binglangjiang buffalo casein [15], have also been shown to be promising resources for producing α-glucosidase inhibitory peptides. Therefore, the screening of α-glucosidase inhibitory peptides from natural food resources offers a promising approach for developing functional, natural alternatives to synthetic α-glucosidase inhibitors for diabetes management.

Industrial hemp is a nondrug type of *Cannabis sativa* L. that is commonly used in the field of fiber production or as an abundant resource for bioactive phytochemicals [16]. The hemp seed has been used as food for a long history due to its favorable nutritional content, including 30–40% dietary fiber, 25–30% lipid, and less than 30% protein [17]. Due to its high protein content, hemp seed has become a renewed resource for bioactive peptide screening and identification. In recent studies, xanthine oxidase inhibitory peptides have been identified from hemp seed protein (HSP) by virtual screening and validated using directional proteolysis by pancreatic elastase [18]. In the elastase hydrolysate of HSP, the peptide DDNPRRFY demonstrated the highest xanthine oxidase inhibitory activity, with an IC_50_ of 2.10 ± 0.06 mg/mL, acting as a mixed inhibitor. Additionally, angiotensin-converting enzyme (ACE) inhibitory peptides, including GVLY, IEE, LGV, and RVR, were also discovered from extensive chemical hydrolysate of HSP using 6 M HCl, confirming that hemp seeds may be a valuable source of hypotensive peptides. In addition, pancreatic lipase inhibitory activity [19], antioxidant activity [20], and immunomodulatory activity [21] have also been observed in the hydrolysate of HSP.

Hypoglycemic peptides from HSP are also reported. For instance, dipeptidyl peptidase IV (DPP-IV) inhibitory peptides were screened from the simulated hydrolysate of HSP based on their binding affinity to DPP-IV, and sixteen potent DPP-IV inhibitory peptides were obtained with IC_50_ values lower than 0.5 mM [22]. As another important target, some α-glucosidase inhibitory peptides have been reported from HSP. For example, the HSP hydrolysate prepared by alkaline protease was found to possess α-glucosidase inhibitory activity, which was further enhanced after graded ethanol precipitation. The hemp protein peptides were suggested to regulate blood glucose and blood lipids in hyperglycemic rats, demonstrating their efficacy for the treatment of diabetes [23]. In another study, the hydrolysate prepared from hemp seed meal with α-glucosidase inhibitory activity prevented intracellular disaccharidase and the transport of glucose [24]. All of this evidence suggests that the HSP-derived peptides are a promising reservoir for the discovery of α-glucosidase inhibitors.

On such a basis, and aiming to discover novel α-glucosidase inhibitory peptides from HSP, the peptide profile of its tryptic hydrolysate was determined after comparing various hydrolysates prepared using different proteases in terms of hydrolysis degree and α-glucosidase inhibitory activity. Through in silico analysis, thirteen peptides were screened out, among which the top three peptides were further investigated using molecular docking and dynamics simulation to elucidate their molecular mechanism of α-glucosidase inhibition.

## 2. Results and Discussion

### 2.1. Protease Screening for HSP Hydrolysis

**Figure 1:**
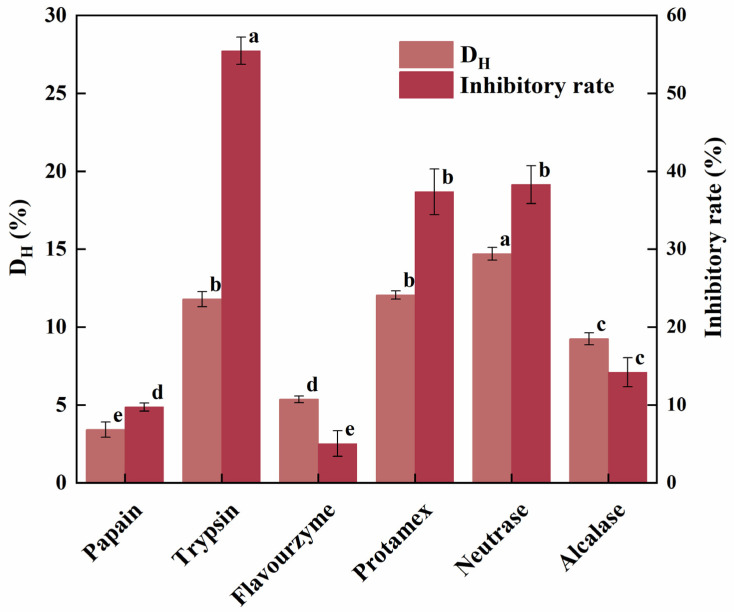
D_H_ and α-glucosidase inhibitory activity of the hydrolysates from HSP. The results were expressed as mean ± S.D. (n = 3), and significant differences (*p* < 0.05) based on Duncan’s test were labeled using superscript letters a, b, c, d, and e on the bars.

In this study, the effects of various proteases on the hydrolysis of HSP were compared in terms of D_H_ and α-glucosidase inhibitory activity of the corresponding hydrolysates (Figure 1). The D_H_ serves as an indicator of the extent of protein cleavage during hydrolysis and is also a key factor that may indirectly reflect the sequence length and molecular weight range of the peptides in the resulting hydrolysate. Overall, the D_H_ of HSP ranged from about 3% to 15%, with Neutrase achieving the highest D_H_ (14.70%), followed by Protamex (12.05%) and trypsin (11.79%). On the other hand, papain exhibited the lowest D_H_, with a value of 3.41%.

Additionally, α-glucosidase inhibitors prevent carbohydrate digestion by competitively inhibiting α-glucosidase residing in the small intestine. Therefore, the α-glucosidase inhibitory rate can also be used for protease screening, aiming at discovery of antidiabetic peptides. In this study, tryptic hydrolysate exhibited the highest inhibitory activity against α-glucosidase, reaching 55.47%, followed by Neutrase (38.28%) and Protamex hydrolysates (37.36%), whereas the flavourzyme hydrolysate exhibited the lowest inhibitory activity (Figure 1). A previous study on the hydrolysis of camellia seed meal has revealed that the α-glucosidase inhibitory rates of the hydrolysates produced by various proteases ranged from 10% to 55%, with the Protamex hydrolysate showing the highest inhibition, followed by trypsin and alkaline protease, and the flavourzyme hydrolysates showing the lowest inhibition [25]. This finding is similar to our results, which may be attributed to the specific terminal groups produced after cleavage, due to the specific cleavage site of each protease for the proteins. For instance, Ibrahim et al. found that an N-terminal hydroxyl-containing residue is critical for α-glucosidase inhibitory activity [26]. Additionally, Stefano et al. reported that peptides containing serine, threonine, tyrosine, lysine, or arginine at the N-terminus, and proline closer to the C-terminus occupied by methionine or alanine, exhibited higher α-glucosidase inhibitory activity [27].

Furthermore, multiple comparisons revealed significant intergroup variations (*p* < 0.01), and Pearson correlation analysis showed a strong positive correlation between D_H_ and α-glucosidase inhibitory rate (r = 0.8125, *p* = 0.0495). This suggests that a higher degree of hydrolysis tends to produce hydrolysates with stronger α-glucosidase inhibition. Based on both of D_H_ and α-glucosidase inhibition, the tryptic hydrolysate of HSP was selected for peptidomics analysis.

### 2.2. Peptidomics Analysis

Nano LC-MS/MS was used to analyze the peptide profile of the tryptic hydrolysate of HSP, resulting in the identification of 424 peptide sequences.

**Figure 2:**
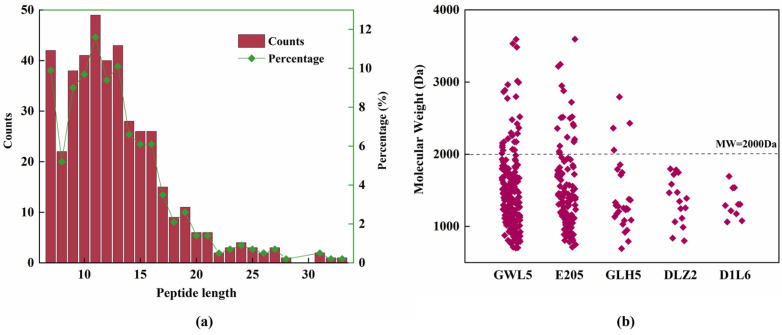
The peptide profile analysis of the tryptic hydrolysate of HSP. (**a**) Distribution of peptide length. (**b**) Molecular weight distribution of the peptides from the top 5 proteins with the most peptides derived. GWL5: A0A7J6GWL5, cupin type-1 domain-containing protein; E205: A0A7J6E205, cupin type-1 domain-containing protein; GLH5: A0A7J6GLH5, cupin type-1 domain-containing protein; DLZ2: A0A7J6DLZ2, cupin type-1 domain-containing protein; D1L6: A0A219D1L6, albumin.

The molecular weights of the peptides ranged from 693 Da to 3595 Da, with sequence lengths varying from 7 to 33 amino acids (Figure 2a). Among the 424 peptides detected, 49 were decapeptides, making up the largest proportion (11.56%), followed by tridecapeptides and heptapeptides. A total of 143 peptides were decapeptides or smaller peptides, accounting for approximately 33.72% of the total peptides. Additionally, 329 peptides were pentadecapeptides or smaller, representing about 80% of the total peptides. No hexapeptides or smaller peptides were detected, possibly due to the relatively low D_H_ of HSP by trypsin.

Regarding the origin of the peptides, most were derived from cupin-type-1 domain-containing proteins, specifically those with UniProt ID A0A7J6GWL5 (GWL5), A0A7J6E205 (E205), A0A7J6GLH5 (GLH5), and A0A7J6DLZ2 (DLZ2), which contributed 209, 122, 27, and 16 peptides, respectively (Figure 2b). Together, these proteins accounted for nearly 90% of the total peptides. GWL5 and E205 are commonly classified as 11S seed storage proteins, often referred to as edestin in hemp seeds, which account for 60–80% of the total hemp seed protein content [11]. This explains why they were the main contributors to the peptides in the hydrolysates. After these four proteins, albumin A0A219D1L6 (D1L6) produced 10 peptides. In hemp seed, albumins are more prevalent than globulins, further demonstrating that the distribution of peptides in this study is consistent with the known protein composition of hemp seed.

Among the total 424 peptide sequences, 68 peptides were carbamidomethylated at cysteine residues, 25 peptides were oxidized at methionine residues, 1 peptide showed N-terminal acetylation, 5 peptides had both carbamidomethylation and oxidation, and 5 peptides had both acetylation and oxidation modifications. After excluding these modified peptides, a total of 323 peptides were used for subsequent activity prediction.

### 2.3. Prediction of Potential Activity of the Peptides in Tryptic Hydrolysates of HSP

In this study, the PeptideRanker program was used to evaluate the potential biological activity of the identified peptides. PeptideRanker utilizes a novel N-to-1 neural network to predict the likelihood that a peptide has biological activity. Any peptide with a score greater than 0.500 is considered to have potential biological activity. Of the 323 peptides evaluated in this study, 47 peptides scored above the threshold of 0.500 (Table 1), with lengths ranging from 7 to 17 amino acids. Among these, there were nine nonapeptides, eight heptapeptides, seven dodecapeptides, five octapeptides, and five undecapeptides. The heptapeptides, octapeptides, and nonapeptides accounted for about a quarter of the total peptides, showing a relatively high proportion of shorter peptides, which tend to have higher bioactivity potential as mentioned before. Previous studies have indicated that short peptides containing phenylalanine and glycine are more likely to exhibit biological activity [13]. However, the results of this study showed that the biological activity, i.e., PeptideRanker score, had little correlation with the phenylalanine and glycine content in the peptides, but it did show a relationship with the length of the peptide sequence (Supplementary Figure S1).

### 2.4. Analysis of Physicochemical Properties of the Peptides

In this study, the physicochemical properties of the 47 peptides with potential biological activity were analyzed (Table 2). The isoelectric points (pI) of these peptides ranged from 0.60 to 11.29. Among them, 29 peptides were acidic (pI < 7.0), whereas 18 were basic (pI > 7.0). The acidic peptides included 8 peptides with pI < 3.0, 8 peptides with pI between 3.0 and 6.0, and 13 peptides with pI between 6.0 and 7.0. Among the basic peptides, 4 peptides were weakly basic (pI between 7.0 and 8.0), 13 peptides were basic (pI between 8.0 and 11.0), and 1 peptide was extremely basci (pI > 11.0). The net charge of these peptides at pH 7.0 ranged from −2.0 to 4.0, showing a good positive correlation with the isoelectric points (*r* = 0.88) (Supplementary Figure S1). However, although isoelectric points are not a significant variable for α-glucosidase inhibition, a net charge of 0.0 or +1.0 was predicted for the highly active peptides [26]. In this study, 30 peptides were selected with a net charge at pH 7.0 of 0.0 or +1.0, accounting for 63.8% of the total, indicating a favorable selection outcome.

**Figure 3:**
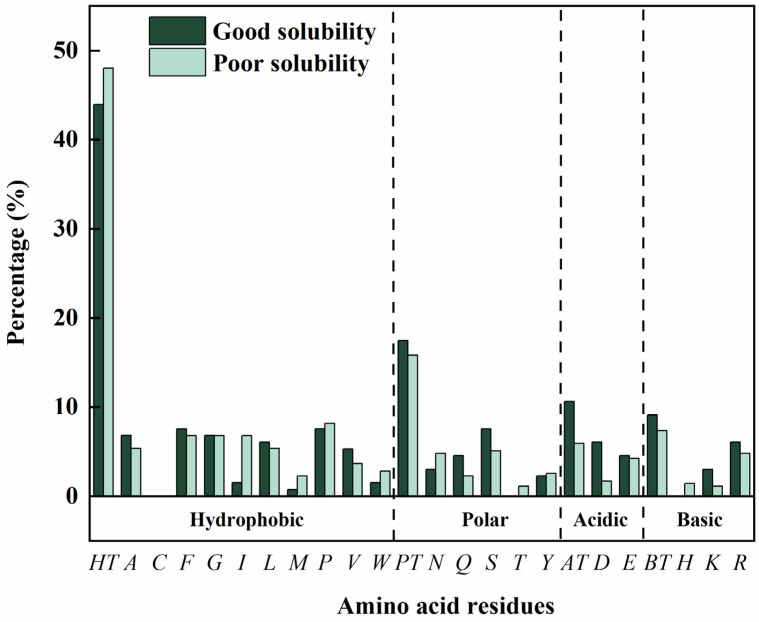
Effect of amino acid residues on water solubility of the peptides. The amino acid residues on abscissa were expressed using one letter code, and the abbreviations were as follows: HT, total percentage of hydrophobic amino acid; PT, total percentage of polar amino acid; AT, total percentage of acidic amino acid; BT, total percentage of basic amino acid.

Furthermore, the water solubility of the peptides was also predicted as it is a crucial factor for their in vivo biological functioning [28]. Among these 47 peptides, 13 peptides exhibited good water solubility, accounting for more than a quarter. Two key factors were generally considered to influence water solubility, namely, the type of amino acid residues and the peptide length [29]. For these reasons, the effect of the amino acid composition on the water solubility was initially analyzed. Generally, more polar amino acids, acidic amino acids and basic amino acids, and less hydrophobic amino acids were observed in the water-soluble peptides (Figure 3). Specifically, as compared with the poorly water-soluble peptides, the contents of the polar amino acid glutarnine, the acidic amino acid aspartic acid and the basic amino acid lysine are significantly higher, and the contents of the hydrophobic amino acid isoleucine and methionine are significantly lower in the water-soluble peptides. Moreover, the presence of arginine or phenylalanine at C-terminus was observed to enhance peptide water solubility, aligning with findings from previous research [30]. On the other hand, no clear relationship was found between sequence length and water solubility in this study, which might be attributed to the fact that most peptide lengths were concentrated within a narrow range and did not vary significantly.

### 2.5. Toxicity and ADMET Property Analysis of the Peptides

Safety is crucial for the potential application of peptides in food, healthcare products, and drugs. Therefore, the ToxinPred program was used to evaluate the toxicity of 13 water-soluble peptides with potential biological activity. ToxinPred uses a support vector machine (SVM) algorithm to predict the potential toxicity of peptides on biological systems and has been widely validated for its reliability [15]. In this study, the SVM scores for all 13 peptides were less than 0 (Table 3), indicating that these peptides are non-toxic.

When used for virtual screening of bioactive agents, the ADMET properties of peptides are crucial [31]. In this study, the ADMET properties of these 13 peptides were assessed using the AdmetSAR program. For distribution, none of the peptides crossed the blood–brain barrier, likely due to their relatively large molecular weights (Table 3). However, for adsorption, 8 out of the 13 peptides were predicted to be absorbable in the human intestinal tract, indicating that most of the peptides could be administered orally (Table 3). Regarding metabolism, the interaction of the peptides with CYP450 2C9, a key enzyme of the CYP450 family, was analyzed. CYP450 family enzymes are important for toxicity and therapeutic efficacy, as they degrade the molecules from circulation in the liver. Thus, inhibition of these enzymes can lead to abnormal biodegradation of the molecules [32]. In this analysis, none of the peptides were found to act as either substrates or inhibitors of CYP450 2C9 (Table 3). Similar results were also observed for other enzymes in the CYP450 family, suggesting a reduced risk of abnormal metabolic interactions.

### 2.6. Molecular Docking

Generally, binding energy plays a crucial role in protein–ligand interactions, as lower binding energy reflects stronger stability of the protein–ligand complex [33]. Therefore, it is commonly used for the rapid screening of inhibitors for specific proteins, including ACE [34], DPP-IV [35], xanthine oxidase [36] and tyrosinase [30], etc. On such a basis, molecular docking was performed to evaluate the binding affinities between the peptides and α-glucosidase, further validating the inhibitory effects of the selected peptides on α-glucosidase (Table 4). In this study, all of the selected peptides exhibited negative binding energies lower than −5.0 kcal/mol, indicating strong and stable interactions with the protein receptor, as demonstrated in previous research [37].

Furthermore, interaction between the peptides and α-glucosidase primarily consisted of hydrogen bonds and hydrophobic interactions, with the number of hydrogen bonds higher than that of hydrophobic interactions. This result aligns with previous studies demonstrating that hydrogen bonds and hydrophobic interactions play a dominant role in α-glucosidase inhibition [38,39].

**Figure 4:**
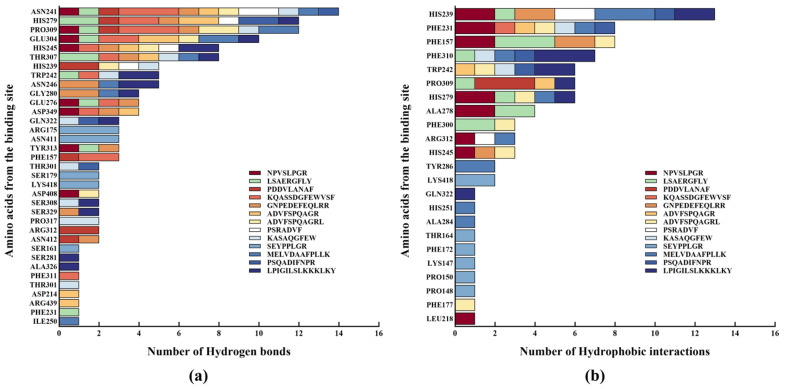
Analysis of the amino acid residues on α-glucosidase involved in (**a**) hydrogen bonding and (**b**) hydrophobic interaction with the peptides in molecular docking.

**Figure 5:**
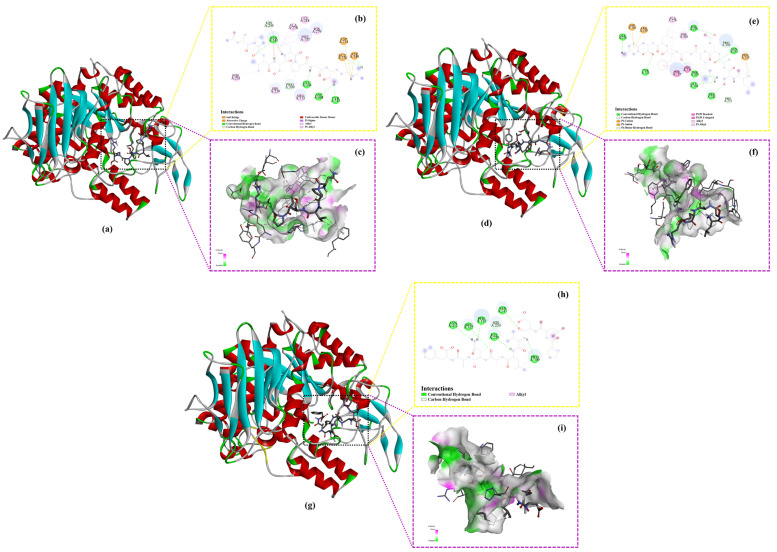
Interaction between the peptides with α-glucosidase: (**a**) 3D structure of the complex NPVSLPGR-α-glucosidase; (**b**) 2D diagram of the interaction between NPVSLPGR and α-glucosidase; (**c**) hydrogen bond surface of the complex NPVSLPGR-α-glucosidase; (**d**) 3D structure of the complex LSAERGFLY-α-glucosidase; (**e**) 2D diagram of the interaction between LSAERGFLY and α-glucosidase; (**f**) hydrogen bond surface of the complex LSAERGFLY-α-glucosidase; (**g**) 3D structure of the complex PDDVLANAF-α-glucosidase; (**h**) 2D diagram of the interaction between PDDVLANAF and α-glucosidase; (**i**) hydrogen bond surface of the complex PDDVLANAF-α-glucosidase.

As shown in the binding site analysis (Figure 4), the key residues on α-glucosidase involved in hydrogen bond formation with the peptides include Asn241, His279, Pro309, Glu304, His245, and Thr307, while the residues responsible for hydrophobic interactions primarily comprise His239, Phe231, Phe157, and Phe310. In *S. cerevisiae* α-glucosidase, the gatekeeping residues, including Phe231, His239, Asn241, His279, Glu304, and Arg312, control the entry and exit of the substrate into the active site [40]. These results suggest that the peptides in this study exert their α-glucosidase inhibitory effect mainly by occupying the entrance of the active site gorge, as further illustrated by the docking conformation in Figure 5.

In addition, a few electrostatic interactions were observed between the peptides and α-glucosidase at specific amino acid residues, mainly including Asp349, Glu276, and Asp214. In the active sites of *S. cerevisiae* α-glucosidase, Asp214, Glu276, and Asp349 form a catalytic triad, where Asp214 acts as a nucleophile, Glu276 serves as a proton donor, and Asp349 stabilizes the substrate’s transition state [40]. Although the number of electrostatic interactions between the peptides and α-glucosidase was fewer than that of hydrogen bonds and hydrophobic interactions, their role cannot be overlooked due to their critical involvement in the interactions with the active sites.

In order to further evaluate the inhibitory effect of the peptides on α-glucosidase, their binding affinities were compared with that of acarbose, a commonly used positive control in the screening of potent α-glucosidase inhibitors. The binding energy of acarbose obtained in this study was −8.1 kcal/mol (Table 4), which was similar to that reported in a previous study (−7.9 kcal/mol) [41]. Compared with acarbose, three peptides, i.e., NPVSLPGR (−8.7 kcal/mol), LSAERGFLY (−8.5 kcal/mol), and PDDVLANAF (−8.4 kcal/mol), were found to have lower binding energies, indicating stronger and more stable binding, and thus, a potentially more potent inhibition on α-glucosidase. As shown in a previous study, compounds derived from the marine macro brown alga *Dictyopteris hoytii*, which exhibited lower binding energies to α-glucosidase, also demonstrated lower IC_50_ values, indicating a positive correlation between binding energy and IC_50_ values [26]. A similar observation was also achieved in this study, further confirming the efficacy of these three α-glucosidase inhibitory peptides.

As illustrated by the binding conformations, all three peptides bind to the active pocket of α-glucosidase through hydrogen bonding, hydrophobic interaction, and electrostatic interaction (except for PDDVLANAF) (Figure 5 and Table 4). Since hydrogen bonding plays a critical role in non-bonding interactions and dominates the interactions between the peptides and α-glucosidase, the hydrogen bond surfaces were analyzed in greater detail (Figure 5c,f,i). Hydrogen bonds comprise hydrogen bond donors (purple) and hydrogen bond acceptors (green). In the hydrogen bond surface, the hydrogen bond donor defines the area with lower electron density in the electronegative atom compared with the hydrogen bond acceptor [42]. In this study, all three peptides exhibited more hydrogen bond acceptor areas than hydrogen bond donor areas.

Based on the findings discussed above, it can be inferred that the peptides NPVSLPGR and LSAERGFLY inhibit α-glucosidase by occupying the entrance of the active site gorge to block the entry of substrates, and binding to active sites to prevent catalysis, while the peptide PDDVLANAF primarily inhibits α-glucosidase by occupying the entrance of the active site gorge.

### 2.7. Molecular Dynamics

In this study, MD simulations were conducted to further elucidate the structural dynamic changes in the complexes formed by the peptides and α-glucosidase. Before the MD simulation, the complex of NPVSLPGR-α-glucosidase was pre-simulated for 200 ns to determine the time required for stabilization (Supplementary Figure S2). The system stabilized after approximately 20 ns, with minimal variation in the average RMSD value (2.77 ± 0.25 Å, from 20 ns to 200 ns). This was not significantly different from the RMSD values observed between 20 ns and 50 ns. Therefore, the MD analyses were conducted for 50 ns for all complexes.

**Figure 6:**
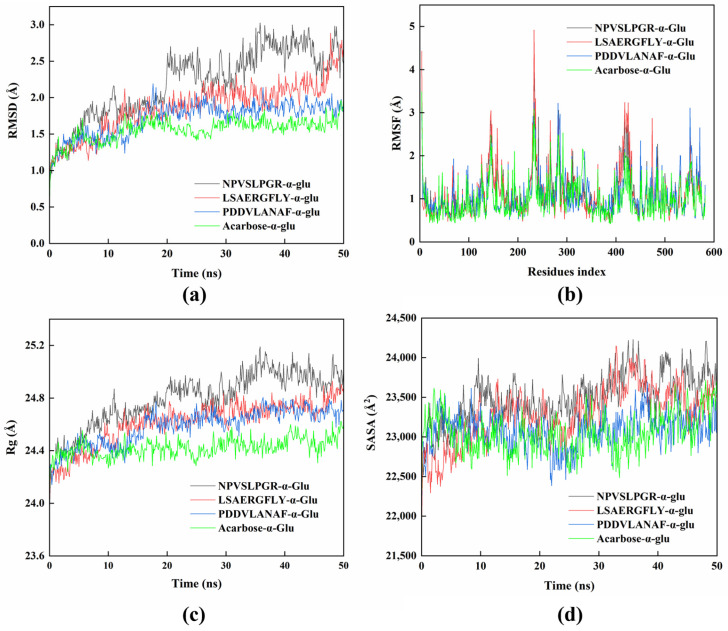
MD simulation (50 ns) of the peptide-α-glucosidase complex. (**a**) Root mean square deviation (RMSD) values and (**b**) root mean square fluctuation (RMSF) values of the complex; (**c**) radius of gyration (Rg), and (**d**) solvent-accessible surface area (SASA) values.

Generally, lower RMSD value indicates a more stable global structure [43]. In this study, the RMSD values showed a slight increase from approximately 1 Å to about 2.5 Å (the complex of NPVSLPGR-α-glucosidase), about 2 Å (the complex of LSAERGFLY-α-glucosidase and PDDVLANAF-α-glucosidase) or about 1.5 Å (the complex of acarbose-α-glucosidase) during the first 20 ns, after which they stabilized with variations less than 0.3 Å, indicating a stable complex formation (Figure 6a). The average RMSD values for the complexes NPVSLPGR-α-glucosidase, LSAERGFLY-α-glucosidase, PDDVLANAF-α-glucosidase, and acarbose-α-glucosidase (from 20 ns to 50 ns) were 2.53 ± 0.23 Å, 2.09 ± 0.20 Å, 1.85 ± 0.08 Å, and 1.62 ± 0.09 Å, respectively, indicating a relatively stable backbone structure of the protein. These results align with previous findings on luteolin-α-glucosidase complex, where the RMSD values of the backbone atoms ranged from 2 to 3 Å [44].

RMSF values reflect the fluctuation degree of the amino acid residues in the receptor protein within the complex. In this study, the average RMSF values for the complexes NPVSLPGR-α-glucosidase, LSAERGFLY-α-glucosidase, PDDVLANAF-α-glucosidase, and acarbose-α-glucosidase were 1.15 ± 0.51 Å, 1.13 ± 0.59 Å, 1.09 ± 0.46 Å, and 1.01 ± 0.44 Å, respectively (Figure 6b), indicating a stable structure during MD simulation. Furthermore, the fluctuation patterns of RMSF were consistent with a previous study on the α-glucosidase inhibitory compounds from *Chlorella minutissima*, which exhibited significant fluctuation at residues 130–170, 220–250, 280–300, and 400–450 [45]. As reported previously, residues 280–300 and 400–450 of the α-glucosidase correspond to the α-helices and the loop regions near the active site, respectively [45]. The increased fluctuation in both regions enhances the flexibility of the entrance pocket residues, thereby facilitating peptide binding at the active site. This observation corroborates the findings from molecular docking.

The Rg value generally determines the compactness of the protein structure, with lower Rg values reflecting a more closely packed protein. The average Rg values for the complexes NPVSLPGR-α-glucosidase (24.79 ± 0.21 Å), LSAERGFLY-α-glucosidase (24.61 ± 0.17 Å), PDDVLANAF-α-glucosidase (24.58 ± 0.14 Å), and acarbose-α-glucosidase (24.43 ± 0.07 Å) were at similar level and varied minimally (Figure 6c). In a previous study on α-glucosidase inhibitory peptides from *Ginkgo biloba* seed cake, the Rg values for the peptide-α-glucosidase complexes ranged from 23.5 to 23.7 Å, which are comparable to the values observed in this study [46].

SASA is commonly used to describe the folding stability of a receptor protein and its surface volume change [47]. In this study, the SASA values for the complexes increased slightly with time (Figure 6d), suggesting that the protein pocket bound by the peptides expanded slightly, resulting in a larger surface area.

## 3. Materials and Methods

### 3.1. Materials and Reagents

HSP with protein content > 70% was provided by Shaanxi Jinrun Biotechnology Co., Ltd. (Xi’an, China). The proteases used in this study include Flavourzyme, Alcalase 2.4L, Protamex, and Neutrase from Novozymes Biotechnology Co., Ltd. (Tianjin, China), trypsin from Sigma-Aldrich (Shanghai, China), and papain from Shanghai Aladin Biotech Co., Ltd. (Shanghai, China). α-Glucosidase from *Saccharomyces cerevisiae* (EC 3.2.1.20) and *p*-nitrophenyl-α-D-glucopyranoside (*p*NPG) were purchased from Sigma-Aldrich (Shanghai, China).

### 3.2. Hydrolysis of HSP

HSP powder was exactly weighed out and thoroughly mixed with a buffer as specified (Supplementary Table S1) in a ratio of 5% (HSP: buffer, *w*/*v*), after which the protease was added at an E/S ratio of 1%. The hydrolysis was carried out for 6 h at an optimal temperature for respective protease (Supplementary Table S1). After hydrolysis, the mixture was boiled for 10 min to stop the reaction, and then centrifuged at 10,000× *g* for 10 min. The supernatant was collected, lyophilized, and stored at −20 °C.

### 3.3. Determination of Degree of Hydrolysis (D_H_)

The D_H_ was determined using the *o*-phthalaldehyde (OPA) assay as described by Zhang et al. [48]. Briefly, OPA working solution was initially prepared by thoroughly mixing 25.0 mL of 100 mM aqueous sodium tetraborate solution, 10.0 mL of 5.0% (*w*/*v*) aqueous sodium dodecyl sulfate solution, 1.0 mL of 40 mg/mL OPA solution in methanol, 0.1 mL of β-mercaptoethanol, and 13.9 mL of water. Subsequently, 50.0 µL of HSP hydrolysate was mixed with 2.0 mL of the OPA working solution and reacted at room temperature for 8 min. After the reaction, the absorbance at 340 nm was measured using a spectrophotometer (L5S, INESA Analytical Instrument Co., LTD, Shanghai, China), and the free amino content was calculated by the detected absorbance against the calibration curve established using glycine–glycine dipeptide as the standard. The D_H_ of the HSP hydrolysate was calculated according to the following formula:

D_H_ (%) = (C_2_ − C_1_)/(C_0_ − C_1_) × 100 (1)

C₁ is the free amino content of HSP before hydrolysis (mol/L); C₂ is the free amino content of HSP after hydrolysis (mol/L); and C₀ is the total amino content of HSP determined by the Kjeldahl method (mol/L).

### 3.4. Determination of α-Glucosidase Inhibitory Activity

The α-glucosidase inhibitory activity of HSP hydrolysates was determined according to the method described by Liu et al. [49]. Briefly, 100.0 µL of sample was mixed with 400.0 µL of α-glucosidase solution (0.5 U/mL) and incubated at 37 °C for 10 min. Subsequently, 50.0 µL of *p*NPG (5 mM) was added to the mixture, thoroughly mixed, and incubated at 37 °C for another 30 min. After the reaction, 100.0 µL of 1 M Na₂CO₃ solution was added to terminate the reaction, and the absorbance at 405 nm was measured. The α-glucosidase inhibitory activity was calculated using the following formula:

α-glucosidase inhibitory rate (%) = (1 − (A_1_ − A_2_)/(A_3_ − A_4_)) × 100 (2)

A₁ refers to the absorbance of the sample, i.e., the HSP hydrolysate; A₂ refers to the absorbance of the sample blank; A₃ refers to the absorbance of the negative control (where the sample is replaced by buffer); and A₄ refers to the absorbance of the negative control blank.

### 3.5. Analysis of Peptide Profile

The peptide profile of the tryptic hydrolysate of HSP was determined based on Nano LC-MS/MS using an Ultimate 3000 system coupled with Q Active™ Hybrid Quadrupole-Orbitrap™ mass spectrometry equipped with ESI Nanospray Source (Thermo Fisher Scientific, Waltham, MA, USA) [30].

### 3.6. Evaluation of Potential Biological Activity of the Peptides

The potential biological activity of the identified peptides was evaluated using the PeptideRanker program (http://distilldeep.ucd.ie/PeptideRanker/ (accessed on 24 April 2024)) with the threshold set at 0.500, since the program was trained at this threshold [50]. Thus, any peptide with a PeptideRanker score higher than 0.500 was deemed bioactive.

### 3.7. Analysis of Physicochemical Properties of the Peptides

The physicochemical properties, including molecular weight, net charge at pH 7.0, isoelectric point, and water solubility, were predicted using an online program Peptide Property Calculator (http://www.innovagen.com/proteomics-tools/ (accessed on 24 April 2024)) for the identified peptides of potential biological activity.

### 3.8. Peptide Toxicity Analysis

The ToxinPred program (https://webs.iiitd.edu.in/raghava/toxinpred/ (accessed on 24 April 2024)) was used to predict the toxicity of the soluble peptides with potential biological activity as identified above [51].

### 3.9. ADMET Evaluation

ADMET properties of the soluble peptides with potential biological activity were analyzed using AdmetSAR (http://lmmd.ecust.edu.cn/admetsar2/ (accessed on 24 April 2024)). The human intestinal absorption (HIA), blood–brain barrier penetration (BBB), and cytochrome P450 (CYP 450) 2D9 interaction were analyzed to reflect the absorption, distribution, and metabolic properties of the peptides [52].

### 3.10. Molecular Docking

Molecular docking was conducted using AutoDock Vina (version 1.1.2), a widely used software for virtual screening of bioactive peptides, despite not being specifically designed for peptide docking [53]. The 3D structure of the peptides for molecular docking was created using Chembio3D Ultra (version 14.0) and saved in MOL2 format. Prior to docking, the 3D structure of the peptides was processed using AutoDock (V 4.2.6). On the other hand, the 3D structure of *Saccharomyces cerevisiae* α-glucosidase, which was the same enzyme used in the in vitro experiments and belongs to the Glycoside Hydrolase (GH) Family 13, was built based on homology modeling using SWISS-MODEL (https://swissmodel.expasy.org/ (accessed on 2 May 2024)), due to the unavailability of the crystal structure of α-glucosidase [45]. The primary sequence of *S. cerevisiae* α-glucosidase (UniProt ID: P53341) was obtained from UniProt (https://www.uniprot.org/ (accessed on 2 May 2024)) and used for homology modeling with the crystal structure of *S. cerevisiae* isomaltase (PDB ID: 3aj7.1A) as a template (GMQE = 0.95). The 3D structure of α-glucosidase obtained from homology modeling was processed by AutoDock before docking to remove water and add hydrogen. Subsequently, site-specific docking between the peptides and α-glucosidase was conducted with the grid centered at center_x = 15.77, center_y = −2.99, and center_z = 17.65 and a grid box size of 60.00 Å × 60.00 Å × 60.00 Å. Conformation with the lowest binding energy was visualized using BIOVIA Discovery Studio Visualizer (V 17.2.0).

### 3.11. Molecular Dynamics (MD) Simulation

MD simulation was conducted based on the molecular docking conformation with the lowest binding energy using YASARA Dynamics (Version 14.10.5) for 50 ns. Simulation was performed using the AMBER14 force field. The simulation cell was defined as “around all atoms” with a size automatically set at 5.00 Å. The environment was set at 298 K and pH 7.4, using 0.9% NaCl and water solvent system. The cell boundaries were set as “periodic”, and the system was energy minimized before MD. All other parameters were used with default settings. In this study, to evaluate the stability of ligand–receptor interactions, the MD simulation results, including changes in root mean square deviation (RMSD), root mean square fluctuation (RMSF), radius of gyration (Rg), and solvent-accessible surface area (SASA), were analyzed.

### 3.12. Statistical Analysis

The results in this study were expressed as mean ± standard deviation (S.D.) and analyzed using Duncan’s multiple range test performed with the IBM SPSS Statistics 20.0 (Chicago, IL, USA) for statistical significance at a level of *p* < 0.05. Additionally, Pearson correlation analysis was conducted using the same software to examine potential relationships between variables.

## 4. Conclusions

In this study, the peptide profile of the tryptic hydrolysate of HSP was determined, as it exhibited favorable D_H_ and the best α-glucosidase inhibitory activity compared to other HSP hydrolysates prepared using different proteases. A total of 424 peptides, with molecular weights ranging from 693 Da to 3595 Da, were identified, originating mainly from four cupin-type-1 domain-containing proteins. Among these peptides, 47 scored above the threshold of 0.500 by PeptideRanker, indicating their potential as bioactive candidates. Based on physiochemical properties, toxicity, and ADMET analysis, 13 peptides were ultimately selected for validation as potential α-glucosidase inhibitors. These 13 peptides interacted with α-glucosidase primarily through hydrogen bonding and hydrophobic interactions at negative binding energies below –6.0 kcal/mol, indicating stable binding, and consequently, strong α-glucosidase inhibition. Among them, three novel peptides, i.e., NPVSLPGR, LSAERGFLY, and PDDVLANAF, displayed binding energies lower than that of the positive control acarbose, suggesting potent α-glucosidase inhibitory activities. The peptides NPVSLPGR and LSAERGFLY inhibited α-glucosidase by both occupying the entrance of the active site gorge to block substrate entry and binding to active sites to prevent catalysis, whereas the peptide PDDVLANAF primarily inhibits α-glucosidase by occupying the entrance of the active site gorge. Furthermore, MD simulation confirmed the stability of the complexes formed between the peptides and the enzyme and corroborated the findings from molecular docking. Based on the findings from computational studies, these peptides demonstrate promising α-glucosidase inhibitory potential and may serve as viable natural alternatives to synthetic inhibitors. However, further validation in vivo and through clinical trials is essential to ensure their effectiveness in real-world applications.

## Figures and Tables

**Table 1:** Activity prediction of the peptides from the tryptic hydrolysate of HSP.

No.	Sequence	PeptideRanker Score	No.	Sequence	PeptideRanker Score
1	NAPMMFY	0.944	25	NPVSLPGR	0.613
2	NLPILRF	0.853	26	DIIAIPAGM	0.608
3	RGLLLPSFLNAPM	0.806	27	AYEPVWAIGTGK	0.605
4	LHFPPHR	0.806	28	KQASSDGFEWVSF	0.603
5	GFEWVSF	0.803	29	EGDIIAIPAGM	0.587
6	PSQADIFNPR	0.779	30	KASAQGFEW	0.583
7	RGLLLPSFL	0.772	31	FHLAGNPHR	0.579
8	PPSGGRFTQIL	0.767	32	AMPDDVLANAF	0.572
9	AGLQFPVGR	0.763	33	PSRADVF	0.570
10	EGDIIAIPAGMAYW	0.755	34	PDDVLANAF	0.558
11	NLPILSFLR	0.748	35	LINPVSLPGRFEPF	0.556
12	SEYPPLGR	0.738	36	EGDIIAIPAGMAY	0.547
13	NLPILSFL	0.732	37	SAQGFEWIAVK	0.546
14	SYNLPILR	0.694	38	GNPEDEFEQLRR	0.532
15	ADVFSPQAGRL	0.693	39	GFEWIAVK	0.525
16	HAQGSGGTIWPF	0.686	40	ASAQGFEWIAVK	0.522
17	IAGNPHQEFPQSMM	0.679	41	ADVFSPQAGR	0.522
18	INPVSLPGRFEPF	0.669	42	HAQGSGGTIWPFGPETR	0.518
19	QASSDGFEWVSF	0.668	43	LINPVSLPGRFEPFY	0.513
20	PAGVAYW	0.660	44	LSAERGFLY	0.509
21	YNLPILR	0.658	45	LINPVSLPGR	0.506
22	LPIGILSLKKKLKY	0.644	46	MELVDAAFPLLK	0.506
23	NNYNLPILR	0.622	47	RIGFLEANPNAF	0.502
24	NLPILSF	0.613			

**Table 2:** Physicochemical properties of the peptides with potential biological activity.

No.	Sequence	pI	Net Charge at pH 7.0	Water Solubility	No.	Sequence	pI	Net Charge at pH 7.0	Water Solubility
1	NAPMMFY	3.24	0.0	Poor	25	NPVSLPGR	10.42	1.0	Good
2	NLPILRF	10.42	1.0	Poor	26	DIIAIPAGM	0.78	−1.0	Poor
3	RGLLLPSFLNAPM	10.55	1.0	Poor	27	AYEPVWAIGTGK	6.84	0.0	Poor
4	LHFPPHR	10.84	1.2	Poor	28	KQASSDGFEWVSF	3.93	−1.0	Good
5	GFEWVSF	0.99	−1.0	Poor	29	EGDIIAIPAGM	0.71	−2.0	Poor
6	PSQADIFNPR	7.08	0.0	Good	30	KASAQGFEW	6.65	0.0	Good
7	RGLLLPSFL	10.55	1.0	Poor	31	FHLAGNPHR	10.59	1.2	Poor
8	PPSGGRFTQIL	11.29	1.0	Poor	32	AMPDDVLANAF	0.61	−2.0	Poor
9	AGLQFPVGR	10.90	1.0	Poor	33	PSRADVF	7.08	0.0	Good
10	EGDIIAIPAGMAYW	0.60	−2.0	Poor	34	PDDVLANAF	0.61	−2.0	Good
11	NLPILSFLR	10.42	1.0	Poor	35	LINPVSLPGRFEPF	6.86	0.0	Poor
12	SEYPPLGR	6.58	0.0	Good	36	EGDIIAIPAGMAY	0.67	−2.0	Poor
13	NLPILSFL	3.21	0.0	Poor	37	SAQGFEWIAVK	6.59	0.0	Poor
14	SYNLPILR	9.57	1.0	Poor	38	GNPEDEFEQLRR	4.04	−2.0	Good
15	ADVFSPQAGRL	6.71	0.0	Good	39	GFEWIAVK	6.85	0.0	Poor
16	HAQGSGGTIWPF	7.56	0.1	Poor	40	ASAQGFEWIAVK	6.91	0.0	Poor
17	IAGNPHQEFPQSMM	5.10	−0.9	Poor	41	ADVFSPQAGR	6.71	0.0	Good
18	INPVSLPGRFEPF	6.87	0.0	Poor	42	HAQGSGGTIWPFGPETR	7.57	0.1	Poor
19	QASSDGFEWVSF	0.70	−2.0	Poor	43	LINPVSLPGRFEPFY	6.81	0.0	Poor
20	PAGVAYW	3.78	0.0	Poor	44	LSAERGFLY	6.81	0.0	Good
21	YNLPILR	9.57	1.0	Poor	45	LINPVSLPGR	10.84	1.0	Poor
22	LPIGILSLKKKLKY	10.77	4.0	Good	46	MELVDAAFPLLK	3.93	−1.0	Good
23	NNYNLPILR	9.41	1.0	Poor	47	RIGFLEANPNAF	6.58	0.0	Poor
24	NLPILSF	3.28	0.0	Poor					

**Table 3:** Toxicity and ADMET analysis of the potential bioactive and water-soluble peptides.

No.	Sequence	Toxicity (SVM Score)	BBB ^1^	HIA ^2^	CYP450 2C9 Substrate ^3^	CYP450 2C9 Inhibitor ^4^
1	PSQADIFNPR	non-toxicity (1.190)	-(0.982)	+(0.743)	-(0.825)	-(0.878)
2	SEYPPLGR	non-toxicity (0.210)	-(0.983)	+(0.637)	-(0.813)	-(0.921)
3	ADVFSPQAGRL	non-toxicity (0.800)	-(0.986)	-(0.539)	-(0.801)	-(0.867)
4	LPIGILSLKKKLKY	non-toxicity (1.320)	-(0.993)	+(0.829)	-(0.866)	-(0.938)
5	NPVSLPGR	non-toxicity (0.890)	-(0.979)	-(0.622)	-(0.832)	-(0.892)
6	KQASSDGFEWVSF	non-toxicity (0.810)	-(0.936)	+(0.802)	-(0.838)	-(0.884)
7	KASAQGFEW	non-toxicity (1.030)	-(0.860)	+(0.765)	-(0.850)	-(0.908)
8	PSRADVF	non-toxicity (0.520)	-(0.920)	-(0.577)	-(0.787)	-(0.911)
9	PDDVLANAF	non-toxicity (0.940)	-(0.901)	+(0.771)	-(0.822)	-(0.928)
10	GNPEDEFEQLRR	non-toxicity (1.160)	-(0.992)	+(0.673)	-(0.770)	-(0.876)
11	ADVFSPQAGR	non-toxicity (0.710)	-(0.986)	-(0.663)	-(0.796)	-(0.892)
12	LSAERGFLY	non-toxicity (0.870)	-(0.953)	+(0.529)	-(0.738)	-(0.839)
13	MELVDAAFPLLK	non-toxicity (1.620)	-(0.990)	+(0.759)	-(0.827)	-(0.910)

^1^ BBB, blood–brain barrier; “-” in the column stands for “not cross BBB”, and + stands for “cross BBB”; the value in the bracket stands for possibility. ^2^ HIA, human intestinal absorption; “-” in the column stands for “no HIA”, and + stands for “HIA”; the value in the bracket stands for possibility. ^3^ “-” in the column stands for “not CYP450 2C9 substrate”, and + stands for “CYP450 2C9 substrate”; the value in the bracket stands for possibility. ^4^ “-” in the column stands for “not CYP450 2C9 inhibitor”, and + stands for “CYP450 2C9 inhibitor”; the value in the bracket stands for possibility.

**Table 4:** Molecular docking results of the selected peptides with α-glucosidase.

Sequence	Binding Energy (kcal/mol)	Amino Acid Residues Involved in Hydrogen Bond (Number of Hydrogen Bonds)	Amino Acid Residues Involved in Electrostatic Interactions (Number of Electrostatic Interactions)	Amino Acid Residues Involved in Hydrophobic Interactions (Number of Hydrophobic Interactions)
NPVSLPGR	−8.7	Asn241, His245, Glu276, Glu304, Pro309, Tyr313, Asp349, Asp408 (8)	Asp214, Glu276, Asp349 (3)	His245, Ala278, Leu218, His279, Phe157, Arg312, His239, Phe231 (13)
LSAERGFLY	−8.5	Phe231, Asn241, Trp242, Glu276, His279, Glu304, Thr307, Pro309, Tyr313 (11)	Asp349, Arg439, Phe310 (3)	Ala278, His279, Phe300, Pro309, Phe310, His239, Phe157 (11)
PDDVLANAF	−8.4	Phe157, His239, Asn241, His279, Pro309, Arg312, Asn412 (9)	Not involved (0)	Pro309 (3)
Acarbose	−8.1	Ile217, Lys262, Asn263, His258, Ala289, Tyr292, Glu293, Ser295 (9)	Not involved (0)	Lys262 (1)
KQASSDGFEWVSF	−8.0	Phe157, Asn241, Trp242, His245, His279, Glu276, Glu304, Thr307, Pro309, Phe311, Asp349 (18)	Asp349, Asp408, Glu276, Asp214 (5)	Phe231 (1)
GNPEDEFEQLRR	−8.0	Asp214, Asn241, His245, Asn246, Glu276, His279, Gly280, Thr307, Pro309, Tyr313, Ser329, Asp349, Asn412, Arg439 (16)	Asp214, Asp349, Glu276, Asp 408 (4)	His245, His239, Phe157 (5)
ADVFSPQAGR	−7.7	Asn241, His245, His279, Glu304, Thr307, Asp349 (8)	Glu276, Asp349 (2)	Phe231, Trp242 Pro309 (3)
ADVFSPQAGRL	−7.6	His239, Asn241, His245, Glu304, Pro309, Asp408 (7)	Asp408 (1)	His279, Trp242, His245, Phe231, Phe177, Phe157, Phe300 (7)
PSRADVF	−7.6	His239, Asn241, His245, His279 (5)	Not involved (0)	His239, Arg312 (3)
KASAQGFEW	−7.1	His239, Asn241, Trp242, Thr301, Thr307, Ser308, Pro309, Pro317, Gln322 (10)	Not involved (0)	Phe231, Phe310, Trp242 (3)
SEYPPLGR	−7.0	Ser161, Arg175, Ser179, Asn411, Lys418 (11)	Glu414 (1)	Pro148, Pro150, Lys147, Phe172, Lys418, Thr164 (7)
MELVDAAFPLLK	−6.9	Asn241, Asn246, Ile250, Gly280, Glu304, Thr307, Pro309 (9)	Not involved (0)	Phe310, His239, His279, Arg312, Phe231, Ala284, Tyr286, His251 (11)
PSQADIFNPR	−6.0	Asn241, His279, Gln322, Thr301 (5)	Glu325 (1)	Trp242, His 239, Phe231, Phe310 (4)
LPIGILSLKKKLKY	−6.0	Trp242, His245, Asn246, His279, Gly280, Ser281, Glu304, Thr307, Ser308, Gln322, Ala326, Ser329 (14)	Glu304, Glu325 (4)	Gln322, Phe310, Pro309, His279, Trp242, His239 (10)

## Data Availability

The datasets used and/or analyzed during the current study are available from the corresponding author upon reasonable request.

## Supplementary Materials

The following supporting information can be downloaded at: https://www.mdpi.com/article/10.3390/ijms26052222/s1.
