# Supplementary material for: Identification and Molecular Mechanism of Novel α-Glucosidase Inhibitory Peptides from the Hydrolysate of Hemp Seed Proteins: Peptidomic Analysis, Molecular Docking, and Dynamics Simulation
Source: Int J Mol Sci. 2025 Feb 28;26(5):2222. doi: 10.3390/ijms26052222 (PMC11899805; doi:10.3390/ijms26052222)
Supplement: Supplementary file 1 [file ijms-26-02222-s001.zip › Supplementary Table S1.pdf]

Supplementary Table S1. Reaction environment for respective proteases.

Proteases	Buffer Used	Optimal pH	Optimal Temperature (℃)
Papain	50 mM phosphate buffer	6	50
Trypsin	50 mM phosphate buffer	8	37
Flavourzyme	50 mM phosphate buffer	6	50
Protamex	50 mM phosphate buffer	7	50
Neutrase	50 mM phosphate buffer	7	40
Alcalase 2.4l	50 mM Glycine-NaOH buffer	10	40
